# Advanced and Metastatic Triple Negative Breast Cancer—Potential New Treatment

**DOI:** 10.3390/cancers17071183

**Published:** 2025-03-31

**Authors:** Monika Pajewska, Olga Partyka, Aleksandra Czerw, Andrzej Deptała, Katarzyna Sygit, Izabela Gąska, Sławomir Porada, Jarosław Drobnik, Piotr Pobrotyn, Urszula Grata-Borkowska, Joanna Furtak-Pobrotyn, Tomasz Banaś, Krzysztof Małecki, Elżbieta Grochans, Szymon Grochans, Anna Maria Cybulska, Daria Schneider-Matyka, Ewa Bandurska, Weronika Ciećko, Natalia Czerw, Michał Marczak, Aleksandra Sierocka, Remigiusz Kozlowski

**Affiliations:** 1 Department of Health Economics and Medical Law, Medical University of Warsaw, 01-445 Warsaw, Poland; 2 Department of Economic and System Analyses, National Institute of Public Health NIH-National Research Institute, 00-791 Warsaw, Poland; 3 Department of Oncology Propaedeutics, Medical University of Warsaw, 01-445 Warsaw, Poland; 4 Faculty of Medicine and Health Sciences, Calisia University, 62-800 Kalisz, Poland; 5 Medical Institute, Jan Grodek State University in Sanok, 38-500 Sanok, Poland; 6 Faculty of Health Sciences and Psychology, Collegium Medicum, University of Rzeszów, 35-310 Rzeszow, Poland; 7 Department of Family Medicine, Faculty of Medicine, Wroclaw Medical University, 51-141 Wroclaw, Poland; 8 Pulsantis Specialist and Rehabilitation Clinic Ltd., 53-238 Wroclaw, Poland; 9 Citodent Dental Center Furtak-Pobrotyn & Company Limited Partnership, 05-220 Olawa, Poland; 10 Department of Radiotherapy, Maria Sklodowska-Curie Institute-Oncology Center, 31-115 Cracow, Poland; 11 Department of Radiotherapy for Children and Adults, University Children’s Hospital of Cracow, 30-663 Cracow, Poland; 12 Department of Nursing, Faculty of Health Sciences, Pomeranian Medical University in Szczecin, 71-210 Szczecin, Poland; 13 Department of Pediatric and Oncological Surgery, Urology and Hand Surgery, Faculty of Medicine and Dentistry, Pomeranian Medical University in Szczecin, 71-252 Szczecin, Poland; 14 Center for Competence Development, Integrated Care and e-Health, Medical University of Gdansk, 80-204 Gdansk, Poland; 15 Students’ Scientific Organization of Cancer Cell Biology, Department of Oncology Propaedeutics, Medical University of Warsaw, 01-445 Warsaw, Poland; 16 Department of Innovation, Merito University in Poznan, 61-895 Poznan, Poland; 17 Department of Management and Logistics in Healthcare, Medical University of Lodz, 90-131 Lodz, Poland

**Keywords:** TNBC, metastatic breast cancer, treatment, advanced breast cancer, new treatment

## Abstract

Globally, breast cancer is both the most common cancer and the most common cause of death related to cancer among women. It is estimated that over 2 million women were diagnosed with breast cancer in 2022 worldwide, while almost 400,000 were diagnosed in the EU. Breast cancer has different histopathological subtypes that require different therapeutic approaches. Triple-negative breast cancer (TNBC) is a type that is considered more aggressive; it occurs in about 10 to 20% of all breast cancer cases. Approximately 40% of women initially diagnosed with TNBC will develop metastases. The objective of this study is to present current clinical trials focused on new treatment of advanced and metastatic TNBC. The study was conducted by searching the clinicaltrials.gov database. Due to the scope of this paper, primary endpoints in the included studies were objective response rate, treatment-emergent adverse events or serious adverse events, progression-free survival, and probability of pathologic complete response. Some of the selected studies were phase I or II RCTs; therefore, we should carefully examine their future results and implications for clinical guidelines.

## Simple Summary

Breast cancer accounts for 1 in 6 cancer deaths worldwide. It is also the most commonly diagnosed cancer among women worldwide. Metastasis is the main cause of death in breast cancer; the 5-year survival rate of patients with metastatic breast cancer (MBC) is still less than 30%. The aim of our study was to present current clinical trials in treatment of metastatic and advanced breast cancer. To identify those studies, a search was performed in the clinicaltrials.gov database. Further studies, particularly interventional and randomized trials, are needed to determine the effectiveness and safety of innovative therapies patients with advanced and metastatic breast cancer.

## 1. Introduction

Globally, breast cancer is both the most common cancer and the most common cause of death related to cancer among women. According to the WHO data, more than 2 million women have been diagnosed with breast cancer (BC) and almost 700,000 deaths have been reported in 2022 [1]. The incidence rate in Europe in the same year was 34.8 per 100,000 women. The highest mortality rates were recorded in Cyprus (45.1 per 100,000 women), followed by Slovakia (44.9 per 100,000 women). In turn, the lowest mortality rate was recorded in Spain (23.3 per 100,000 women) [2]. In Poland, according to data from the National Cancer Registry, the crude death rate for women was 32.64 in 2021 [3]. Although breast cancer mainly affects women, it can also occur in men [4,5].

There are several subtypes of breast cancer: luminal A, luminal B, HER2-positive, and triple-negative (TNBC) [6,7]. Differences underlying gene expression patterns between cancer subtypes reflect fundamental differences between cancers at the molecular level [7,8]. Research suggests that breast cancers with different biological characteristics have different activities that result in different responses to treatment. Considering therapeutic decision-making, it is important to appropriately group breast cancer into clinically relevant subtypes. Luminal tumors show expression of hormone receptors [9]. Luminal A breast cancer is diagnosed in 50–60% of all breast cancer cases. Compared to the other subtypes, it is less aggressive and more susceptible to treatment. Luminal B subtype is much less common and is estimated to account for approximately 10% of breast cancer cases [10]. This subtype rarely shows expression of the HER2 protein, which can accelerate the growth of cancer cells [11]. As a result, luminal B cancer is usually more aggressive and has a higher stage of advancement and worse prognosis than luminal A cancer [12,13,14].

Tumors with overexpression of human epidermal growth factor receptor-2 (HER2) constitute approximately 20% of all breast cancer cases [15]. They are identified by immunostaining or fluorescence in-situ hybridization (FISH). Tumors with HER2 overexpression are most often grade III, but the prognosis in this subtype has improved after the introduction of anti-HER2 therapy because these tumors are sensitive to neoadjuvant chemotherapy based on anthracyclines and taxanes.

In triple-negative breast cancer (TNBC), there is no expression of estrogen (ER), progesterone (PR), and HER2 receptors. TNBC is considered an aggressive subtype; it is estimated that 5-year survival varies from 4% to 20% [16]. There is currently no standard targeted therapy for this subtype. Standard treatments include surgery and prior neoadjuvant and adjuvant chemotherapy [16].

Factors such as younger age at first pregnancy and having several children have a protective effect in the luminal A subtype, while in the case of triple-negative cancer they increase the risk of the disease [17]. This subtype is associated with lower survival and an increased risk of relapse, and is additionally characterized by faster growth and metastases, most often to the brain, lungs, and liver [18]. Patients with TNBC have a significantly shorter survival period, having experienced distant metastases, and the disease is most often detected at an advanced stage. TNBC, more often than other subtypes, is associated with hereditary factors [19]. It is estimated that 35% of TNBC patients are carriers of BRCA1 gene mutations and 8% of patients are carriers of BRCA2 mutations [19].

The most dangerous feature of cancer is the spread of cancer to distant organs (metastases). Metastatic breast cancer (MBC) begins in the breast and spreads to nearby lymph nodes as well as other more distant sites in the body [20]. Of all breast cancer patients, up to 15% develop aggressive disease, leading to the spread of the cancer to other organs within 3 years of the development of the primary tumor. The mechanism of metastatic formation is complicated. It involves numerous cellular processes, such as tumor division, invasion, avoidance of immune surveillance, and regulation of the tissue environment [21]. It was found that after metastasis, breast cancer cells are less sensitive to chemotherapy, and in patients with stage IV breast cancer, the 5-year survival rate ranges from several to several dozen percent [22,23]. Depending on the subtype of breast cancer, the prognosis, occurrence and types of metastases, and overall survival vary [24].

Metastases may be a target for treatment or an indicator of disease progression. Targeted therapies include drugs for which the mechanism of action is based on, among other things, interfering with the proliferation and survival of cancer cells [25]. Targeted treatment for MBC is selected based on several factors i.e., presence hormone receptors, HER-2, cancer recurrence, and the presence and location of metastasis [26]. Current treatment strategies for metastatic breast cancer include systemic therapy (i.e., hormone therapy, chemotherapy, and targeted therapy) and local therapy. The choice of first-line treatment for metastatic cancer is based on tumor-related factors (e.g., tumor subtype) and a number of disease-related factors (e.g., disease-free period, location of recurrence, prior treatment), as well as patient-related factors (e.g., overall physical condition or comorbidities) [26]. According to ESMO recommendations, therapeutic decisions should not be made based on the patient’s age; a multidisciplinary approach is recommended, considering comorbidities and patient’s preferences [26]. MBC is an incurable disease, but implementing appropriate therapeutic strategies can prolong survival. In most cases of TNBC, chemotherapy is recommended as standard treatment. From the perspective of treatment effectiveness, it is important to determine the PD-L1 (Programmed Death Ligand 1) status and mutations within BCRA1/2 [26].

In operable TNBC tumors, the first line of treatment includes surgery followed by adjuvant chemotherapy

The objective of this study is to present currently ongoing clinical trials on possible methods of treating advanced and metastatic triple negative breast cancer.

## 2. Materials and Methods

**Figure 1:**
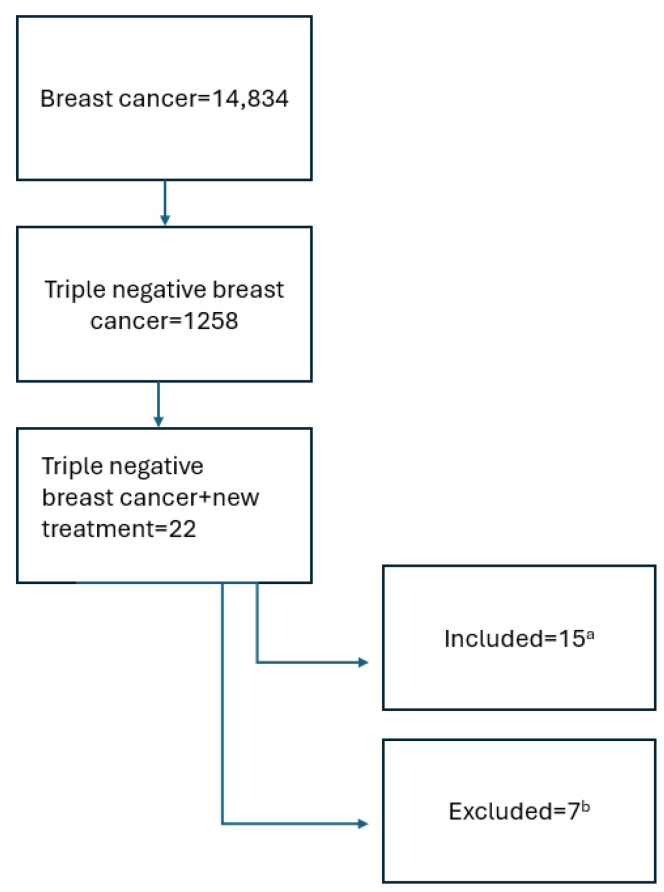
Search scheme. ^a^ the review included studies actively ongoing, recruiting, or completed according to the protocol, with available results. ^b^ terminated studies, observational, and interventional studies where inclusion criteria did not directly concern patients with advanced and metastatic TNBC or intervention did not include new therapies were excluded.

This publication was based on a search in the: https://www.clinicaltrials.gov/search?cond=Triple%20Negative%20Breast%20Cancer&intr=new%20treatment&page=1&limit=50 (accessed on 30 January 2025). The keywords “breast cancer”, “triple negative breast cancer”, “treatment”, and “new treatment” were used. Inclusion criteria included active studies recruiting, active, not recruiting, or completed according to the protocol. Studies that were terminated were excluded. Interventional and observational studies that started in the period from 1 January 2019 to 1 January 2025 were included. The process of qualifying studies for analysis is presented in Figure 1 below.

## 3. Results

Detailed search results are presented in Table 1.

In total, fifteen studies were included in the analysis; fourteen were interventional studies and one was observational. The size of the study population varied and ranged from 9 to 5000 participants. The main endpoints included in our review were objective response rate, treatment-emergent adverse events or serious adverse events, progression-free survival, and probability of pathologic complete response. In the research of new treatment of advanced and metastatic TNBC, parameters such as objective response rate and progression-free survival are important.

The methods of treatment used in the studies presented in Table 1 can be divided into following groups: antibody-drug conjugate, PARP inhibitors, immune checkpoint inhibitors (ICIs) in monotherapy and in combination with AKT inhibitors and chemotherapy, AKT inhibitors, PD-1 inhibitors, and oncolytic virus treatment. Standard treatment for TNBC includes neoadjuvant chemotherapy and surgery followed by adjuvant treatment based on the characteristics of tumor and patients’ status. Detailed treatment options and guidelines are also mentioned in Section 4.

## 4. Discussion

TNBC is the most difficult type of breast cancer to treat, therefore further research is required to develop new therapeutic solutions that will significantly improve the prognosis in this group of patients. The IMpassion130 study evaluated atezolizumab (an anti-PD-L1 monoclonal antibody) in combination with nab-paclitaxel, while the IMpassion131 study evaluated atezolizumab in combination with paclitaxel [42]. In the IM-passion130 study, defined primary endpoints included progression-free survival (PFS) and overall survival (OS) [43]. In the intention-to-treat (ITT) patient group, atezolizumab provided a median PFS benefit of 7.2 months compared to 5.5 months in the placebo plus nab-paclitaxel group (HR = 0.8, 0.95% CI 0.69–0.92; *p* = 0.002). In the PD-L1 positive group, the median PFS was 7.5 months with atezolizumab compared to 5 months in the placebo plus nab-paclitaxel group (HR = 0.62, 95% CI 0.49–0.78; *p* < 0.001). Atezolizumab was approved by the Food and Drug Administration (FDA) for the treatment of locally advanced or metastatic PD-L1-positive TNBC. Meanwhile, in the IMpassion131 study, in the primary PFS analysis, the addition of atezolizumab to paclitaxel did not show improvement in PFS in the PD-L1-positive population (HR = 0.82, 95%, CI 0.60–1.12; *p* = 0.20; median PFS was 6 months in the atezolizumab-paclitaxel group compared with 5.7 months in the placebo-paclitaxel group). Despite the unsatisfactory results of the IMpassion131 study, some patients benefited from atezolizumab/paclitaxel combination therapy [43]. It should be mentioned that the manufacturer of atezolizumab has withdrawn the indication for the use of atezolizumab plus chemotherapy in the treatment of adult patients with unresectable locally advanced or metastatic TNBC in the USA following an extended analysis carried out by the FDA [44].

In the KEYNOTE-355 trial, the addition of pembrolizumab to platinum-based chemotherapy in patients with advanced TNBC who had PD-L1 expression and combined positive score (CPS) was assessed. Patients were divided into groups based on their CPS score [45]. In patients with CPS of 10 or more the median OS was 23 months in the pembrolizumab–chemotherapy group and 16.1 months in the placebo–chemotherapy group (HR for death, 0.73; 95% CI 0.55 to 0.95; two-sided *p* = 0.0185). In the group of patients with CPS of 1 or more, the median OS was 17.6 and 16.0 months in the two groups, respectively (HR 0.86; 95% CI, 0.72 to 1.04; two-sided *p* = 0.1125). In 2020, the FDA granted accelerated approval to pembrolizumab combined with chemotherapy for the treatment of patients with metastatic or locally unresectable TNBC whose tumor has PD-L1 expression [46].

Durvalumab, a PD-L1 inhibitor, has shown promising results in the treatment of bladder and lung cancers [47,48]. BEGONIA phase I/II trial showed promising results in treatment with datopotamab deruxtecan plus durvalumab in patients with unresectable or metastatic TNBC. Preliminary results showed a manageable safety profile and lasting response rates [49].

The CREATE-X study presented the effectiveness of adjuvant capecitabine in patients with residual disease after standard neoadjuvant chemotherapy. The ipatasertib study showed promising results. It was noticed that continuous administration of ipatasertib in combination with chemotherapy had a good safety profile and good tolerance [50].

The randomized phase III EMBRACA study compared the safety and effectiveness of talazoparib with chemotherapy in patients with locally advanced and/or metastatic breast cancer, HER-2 negative, with BRCA1/2 mutations. The study showed that talazoparib had better results in terms of PFS compared to standard chemotherapy. Based on those results, talazoparib was approved by the FDA [51].

Based on data from the Cancer Genome Atlas, it was shown that the most frequently altered genomic factors in TNBC include the phosphatidylinositol 3-kinase-AKT-mTOR signaling pathway [52]. Ipatasertib is a selective, small molecule, ATP-competitive AKT inhibitor that preferentially targets active phosphorylated Akt (pAkt) and is potent in cell lines that have demonstrated Akt activation. In the LOTUS study, the combination of paclitaxel and ipatasertib showed a moderate improvement in PFS in patients with TNBC [53].

Currently, according to the guidelines for patients with TNBC, chemotherapy is recommended as standard treatment [26]. In the case of positive PD-L1, therapy with pembrolizumab in combination with chemotherapy is recommended. In the event of a BRCA1/2 mutation, platinum-based chemotherapy (if it has not been used before) or targeted therapy based on PARP inhibitors is recommended [54].

In the study by Li et al., the analysis was carried out on the data of nearly 160,000 patients who were diagnosed with breast cancer between 2010 and 2012 [55]. The study focused on comparison of OS and cause-specific survival for patients with TNBC and non-TNBC cancer. Patients with TNBC had worse overall survival and cause-specific survival at each stage and substage in univariate and multivariate analyses adjusting for age, race, tumor stage, and surgical and radiotherapy treatment in comparison with non-TNBC patients. A study by Lindman et al. investigated long-term treatment patterns as well as OS in a cohort of patients with MBC stratified by subtype in standard clinical practice and the correlation with current treatment guidelines [56]. Patients with TNBC had the lowest 5-year survival rate of 7% compared to the HER2+/luminal subtype, which was 46% and 29% in the luminal B subtype, respectively. This indicates the need to search for new therapies that will improve prognosis and OS in the group of patients with TNBC.

The ability of cancer cells to evade the immune system has been a significant difficulty in cancer treatment. Oncolytic viruses show promising results in that area; however, according to preliminary reports, viral therapy should be combined with other treatment methods such as chemotherapy, immunotherapy, or targeted therapies. The study No. 9 presented in Table 1 will determine safety, dosage, and objective response rate for a novel chimeric orthopoxvirus in monotherapy or in combination with pembrolizumab in patients with metastatic or advanced solid tumors [35,57].

## 5. Conclusions

Currently, there is ongoing research to develop therapies that might have a positive impact on OS and PFS in patients with advanced TNBC. Patients with this subtype have a much shorter OS, have experienced distant metastases, the disease is most often diagnosed at an advanced stage, and remains incurable. Some novel therapies show promising results, such as durvalumab or oncolytic virus treatment. Oncolytic virus therapies might become an effective therapy in this type of cancer. Since some of the selected studies were phase I or II RCTs, we should carefully examine their future results and implications for clinical guidelines in TNBC.

## Figures and Tables

**Table 1:** Current studies for TNBC started from 1 January 2019 to 1 January 2025.

No.	Study Title	Status	Treatment *	Study Type	Primary Outcome Measures **	Enrollement
1	A Study to Explore Safety, Tolerability, Pharmacokinetics, and Anti-tumor Activity of Novel Therapeutics in Patients with Early Relapsed Metastatic Triple-negative Breast Cancer (COMPASS-TNBC) [27]	Recruiting	Datopotamab Deruxtecan (Dato-DXd)	Interventional	Objective response rate (ORR) at 6 months. Part 1&2_Progression free survival (PFS)	50
2	QL1706 Plus Chemotherapy as Neoadjuvant Therapy in Early High-Risk TNBC Breast Cancer (QUEEN-Dream) [28]	Not yet recruiting	Bispecific antibody (bsAb) targeting PD-1 and CLTA-4 Drug: Albumin-bound paclitaxel and Carboplatin	Interventional	Total Pathological complete response (tpCR) rate using the definition of ypT0/Tis, N0	73
3	A Study on the Efficacy and Safety of Oral All-trans Retinoic Acid Combined with Toripalimab in TNBC [29].	Not yet recruiting	ATRA and Toripalimab	Interventional	Objective Response Rate (ORR) Progression-Free Survival (PFS) Duration of Response (DOR)	32
4	Phase 1/2 Study of Intratumoral Injection of STX-001 in Advanced Solid Tumors as Monotherapy or in Combination with Pembrolizumab [30]	Recruiting	STX-001 and Keytruda^®^	Interventional	Number and nature of dose-limiting toxicities (DLTs), treatment-emergent adverse events (TEAEs) and serious adverse events (SAEs) in patients with advanced solid tumors. Occurrence of changes from baseline in patients’ clinical safety laboratory values and vital signs to assess the safety and tolerability of STX-001. Assessment of pharmacokinetics in patients dosed with STX-001	108
5	Biomarkers of Efficacy and Tolerability of Sacituzumab-Govitecan in the Treatment of Patients with Triple-negative Breast Cancer in the Metastatic Phase: Prospective Multicenter Real-world Study (BIO-PROSA) [31]	Recruiting	sacituzumab govitecan SG	Observational	Biomolecular investigations conducted on multiple platforms PFS in patients. Tolerability of the treatment	60
6	A Study of Tetrathiomolybdate (TM) Plus Capecitabine [32]	Recruiting	Tetrathiomolybdate, Capecitabine, Pembrolizumab	Interventional	Phase 1b: To establish the safety of the combination of adjuvant tetrathiomolybdate with capecitabine and pembrolizumab by the number of dose limiting toxicities Phase 2: Distant relapse-free survival (DRFS) between TM and capecitabine versus capecitabine as measured with the STEEP system Phase 1b: Distant Relapse free survival (DRFS) between TM, capecitabine and pembrolizumab versus capecitabine and pembrolizumab as measured with the STEEP system	204
7	PIK3CA/PTEN-altered Advanced Breast Cancer Treated with MEN1611 Monotherapy or in Combination with Eribulin (SABINA) [33]	Recruiting	MEN1611 and Eribulin	Interventional	To assess the efficacy of MEN1611 in combination with eribulin as determined by the clinical benefit rate (CBR). To determine the efficacy of MEN1611 in combination with eribulin defined as ORR of patients. To determine the efficacy of MEN1611 in combination with eribulin defined as Time To Response (TTR).	14
8	Novel Neoadjuvant and Adjuvant Strategy for Germline BRCA 1/2 Mutated Triple Negative Breast Cancer [34]	Recruiting	Pembrolizumab, Paclitaxel, Carboplatin, Olaparib	Interventional	Pathological Complete Response (pCR) Rate (ypT0/TisypN0) Residual Cancer Burden 0/1 Pathological Complete Response (pCR) Rate (ypT0/is)	23
9	Oncolytic Virus CF33-expressing hNIS/Anti-PD-L1 Antibody [35]	Active, not recruiting	Oncolytic Virus CF33-expressing hNIS/Anti-PD-L1 Antibody	Interventional	Incidence of adverse events	9
10	A Study of Radiation Therapy with Pembrolizumab and Olaparib or Other Radiosensitizers in Women Who Have Triple-Negative or Hormone-Receptor Positive/Her2 Negative Breast Cancer [36]	Recruiting	Pembrolizumab, Olaparib and radiation	Interventional	Overall Response Rate (ORR)	34
11	Study of Safety and Efficacy of DKY709 Alone or in Combination with PDR001 in Patients with Advanced Solid Tumors [37]	Active, not recruiting	DKY709 (Novel immunomodulatory agent)	Interventional	Incidence and severity of AEs and SAEs incidence of Dose Limiting Toxicities (DLTs)	99
12	A Study of Novel Anti-cancer Agents in Patients with Metastatic Triple Negative Breast Cancer (BEGONIA) [38]	Active, not recruiting	Durvalumab, Capivasertib, Oleclumab, Paclitaxel, Trastuzumab deruxtecan, Datopotamab deruxtecan	Interventional	Incidence of adverse events Assessment of safety and tolerability of each treatment arm	243
13	A Multi-Center, Open-Label Study of Fruquintinib in Solid Tumors and Colorectal, and Breast Cancer [39]	Completed	Fruquintinib (HMPL-013)	Interventional	Number of Participants with Dose-limiting Toxicities (DLTs) Number of Participants with Treatment-emergent Adverse Events (TEAEs) and Serious TEAEs	129
14	Study of Cabozantinib in Combination with Atezolizumab to Subjects with Locally Advanced or Metastatic Solid Tumors [40]	Active, not recruiting	Cabozantinib, atezolizumab	Interventional	MTD/Recommended Dose Objective Response Rate (ORR)	1732
15	I-SPY TRIAL: Neoadjuvant and Personalized Adaptive Novel Agents to Treat Breast Cancer (I-SPY) [41]	Recruiting	Pertuzumab, Trastuzumab, Veliparib, Cemiplimab	Interventional	Probability of pathologic complete response (pCR)	5000

* if more than three drugs are tested in one trial, the main three are listed. ** most critical primary outcomes, e.g. PFS, OS, or others.

## Data Availability

No new data were created during preparation of this manuscript.
